# The effectiveness of graphic health warnings on tobacco products: a systematic review on perceived harm and quit intentions

**DOI:** 10.1186/s12889-021-10810-z

**Published:** 2021-05-20

**Authors:** Bo Pang, Pamela Saleme, Tori Seydel, Jeawon Kim, Kathy Knox, Sharyn Rundle-Thiele

**Affiliations:** Social marketing @ Griffith, Department of Marketing, Griffith University, 170 Kessels Road, Nathan, QLD 4111 Australia

**Keywords:** Tobacco, Graphic health warning, Systematic review

## Abstract

**Background:**

Examination of the format and framing of the graphic health warnings (GHWs) on tobacco products and their impact on tobacco cessation has received increasing attention. This review focused on systematically identifying and synthesizing evidence of longitudinal studies that evaluate different GHW formats and specifically considered GHW influence on perceived risk of tobacco use and quit intentions.

**Methods:**

Ten databases were systematically searched for relevant records in December 2017 and again in September 2019. Thirty-five longitudinal studies were identified and analyzed in terms of the formatting of GHWs and the outcomes of perceived risk and quit intentions. Quality assessment of all studies was conducted.

**Results:**

This review found graphics exceeding 50% of packs were the most common ratio for GHWs, and identified an ongoing reliance on negatively framed messages and limited source attribution. Perceived harms and quit intentions were increased by GHWs. However, wear-out effects were observed regardless of GHW format indicating the length of time warnings are present in market warrants ongoing research attention to identify wear out points. Quit intentions and perceived harm were also combined into a cognitive response measure, limiting the evaluation of the effects of each GHW format variables in those cases. In addition, alternative GHW package inserts were found to be a complimentary approach to traditional GHWs.

**Conclusions:**

This review demonstrated the role of GHWs on increasing quit intentions and perceptions of health risks by evaluating quality-assessed longitudinal research designs. The findings of this study recommend testing alternate GHW formats that communicate quit benefits and objective methodologies to extend beyond self-report.

**Supplementary Information:**

The online version contains supplementary material available at 10.1186/s12889-021-10810-z.

## Background

Understanding cognitive reactions after exposure to graphic health warnings (GHWs), is crucial when considering the design and format of GHWs. This review focuses on perceived harm reactions that are operationalized in the literature as thinking about the risks of smoking [1], perceived likelihood of harm from smoking [2] and identifying that smoking causes tobacco related diseases [3], as well as quitting intentions, that are measured as the intention to quit smoking in a certain period of time (i.e. next week) [4] or the extent to which health warning labels make the person more likely to quit smoking [5].

The literature offers theoretical explanations of health risk communications that explain GHWs constructs (perceived risk, cessation intention, smoking behavior) and their relationships [6]. For instance, the extended parallel process model (EPPM) indicates that if a high level of severity from engaging in certain unhealthy behaviors was perceived, individuals will be motivated to behave in order to avoid such risks [7]. Similarly, if individuals hold the belief that they are capable of changing their behavior (perceived self-efficacy) as well as their risk of negative outcomes (perceived response efficacy), the healthier behavior will be motivated to happen. Thus, the EPPM suggests that GHWs messaging that aims to increase smokers’ motivation to quit should include convey messages that contain both threat and efficacy [6].

The WHO Framework Convention on Tobacco Control (FCTC) specifies the importance of labelling health warnings describing the harmful effects of tobacco use in Article 11 [8] but did not provide detailed instructions on the design and format of GHWs apart from generic descriptions of being “large, clear, visible and legible” (p. 10). Depending on the design and format of GHWs, they can have varying effects on cognitive reactions (including perceived risks and quit intentions). The main GHW variable evaluated is format, which includes text vs. graphic warning (or a combination of both), size of the warning on package (i.e. 50%), location (i.e. front of the package) and color. A recent study indicated that a combined disclosure format (text with low/high emotion images) increased risk perceptions and quit intentions relative to text-only [9]. Other evidence suggests that emotionally evocative images depicting hazards (i.e. GHWs proposed by the US FDA) are not effective in communicating wider smoking risks when compared to text featuring irrelevant pictures (e.g. depictions of a car accident), or text-only [10].

Other contradictions are evident for message framing. Positive versus negative framing of tobacco warning messages have been recently examined, contrasting the effects of GHWs communicating the harmful effects of smoking (negative framing) or the benefits of quitting (positive framing). Studies that investigated text message framing effects of tobacco health warnings have found greater efficacy when messages are framed in the negative [11, 12]. However, others identify support for the use of positive message framing when targeting illness prevention behaviors including smoking cessation [13] and yet others have identified reactance to negative messages (fear appeals) for smoker groups [14]. Examination of a wider evidence base for message framing indicates support for the effectiveness of positively framed messages to invoke behavioral change. Examination of 14 direct tests of positive versus negative appeals indicate that 11 studies offer evidence that positive appeals are stronger when compared to negative approaches [14–24]. Hence there remains some uncertainty regarding the role of alternate message framing formats that may further enhance the effectiveness of GHWs. A systematic and comprehensive review of message framing and GHW format is required.

Recent literature reviews have reported the effects of strengthened GHW formats (i.e. larger in size and message) on knowledge and attitudes, attention [25], active smoker’s behavior [26], and young adults including adolescents [27, 28]. Noar et al’s review has a similar focus on longitudinal studies and examines several of the outcomes of interest in the current study. Yet, there is no recent systematic review that has examined the impact of different formats of GHWs on communicating smoking health risks and quit intentions. Because of the paucity of evidence from independent sources, it is important to look at evidence from peer-reviewed journals reporting empirical data. Therefore, this review aims to systematically identify and synthesize evidence from longitudinal studies that have examined GHW formats and their effect on perceived risk of tobacco use and quit intentions of both smokers and non-smokers. The research question of this review is:

*What warning and/or disclosure formats are optimal for communicating the risk of smoking on health and quit intentions?*

## Method

The PRISMA guidelines [29] were followed to conduct the systematic search in order to ensure completeness and transparency.

### Key search terms

The following search terms were used for this review:

tobacco OR cigar* OR bidis OR beedi OR e-cigar* OR heat-not-burn OR shisha OR “heat not burn” OR “roll your own” OR roll-your-own

*AND.*

warning* OR pictorial OR graphic OR packag*

*AND.*

intervention* OR evaluation OR trial OR campaign* OR program* OR experiment* OR effect* OR impact*

The asterisk allowed for the inclusion of term variations (e.g., singular vs plural).

### Databases

Ten databases were systematically searched by three researchers (J.K., T.S., P.S.) for relevant records in December 2017 and again in September 2019. Database names and the number of records retrieved from each database are shown in Table 1. Variation in the number of records retrieved from the different databases is explained by the size and the subject specialization of each database.

**Table 1:** Number of records retrieved by database

Database	Number of records retrieved
Cochrane	182
EBSCO (All Databases)	234
Ovid (All Databases)	984
PubMed	313
Web of Science (All Databases)	621
Embase	783
ProQuest (All Databases)	320
Australasian Medical Index (via Informit)	0
Emerald	72
Grand total	**3509**
Total after duplicates removed	**1359**

The combined total of records downloaded from all databases was 3430. All downloaded records were imported into EndNote X9. From the initial records collected, 2071 duplicate records were removed (first by EndNote, then by reviewers T.S. and P.S.), leaving 1359 unique sources.

All downloaded records were imported into Endnote X9. After duplications were removed, two reviewers (T.S., P.S.) reviewed titles and abstracts of the remaining papers were reviewed for eligibility. Any papers that were

- not peer-reviewed (e.g., newspapers, theses, or conference proceedings);
- not written in English;
- not tobacco focused;
- not GHW focused;
- systematic literature reviews;
- cross-sectional studies with only one time point

were excluded.

Longitudinal studies with empirical data that consisted of the following information were included:

- Published in peer-review journals;
- Published between 2008 and 2019, only studies that were published in the past 10 y were included to ensure the recency of this review;
- Reporting detailed information on the formats of GHWs;
- Reporting on outcomes pertaining to either: communicating the risk of smoking on health, or the benefits of quitting, or quit intention.

Selected studies were qualified for inclusion considering the operationalization of perceived risk and quit intentions, given the diversity of measurements for each behavioral outcome. Perceived risks were operationalized as thinking about the risks of smoking, perceived likelihood of harm from smoking and identifying that smoking causes tobacco related diseases. Quit intentions, were operationalized as the intention to quit smoking in a certain period of time or the extent to which health warning labels make the person more likely to quit smoking.

### Data extraction and coding framework

A coding framework was developed to enable a standardized method for extraction of the following information from qualified records, as shown in Table 2.

- Bibliographic information, such as authors, title, and publication year;
- Study characteristics, such as study location, study design, and sample size;
- Formats of GHWs, such as the size and location of graphics;
- Outcome evaluation, such as the effectiveness of GHWs on communicating the risk of smoking on health and the benefits of quitting, as well as quit intentions.

**Table 2:** Coding framework

Construct	Code(s)
**A. Bibliographic Information**	
Authors(s)	As stated in article
Title	As stated in article
Publication year	As stated in article
**B. Study Characteristics**	
Study design	1. Randomized Controlled Trial (RCT) or Controlled Clinical Trial (CCT)
	2. Quasi Experiment (two or more groups pre and post)
	3. Cohort (one group pre and post)
	4. Interrupted time series (or Longitudinal)
Location	Country, Region
Study aim(s)	As stated in article
Product	cigarette, e-cigar, shisha, beedis, roll your own, etc.
GHWs disclosure formats - Graphics	***Location*** i. Both the front and back ii. On principal display areas (top, bottom) iii. Package inserts iv. Others ***Size*** v. 51% or more vi. Between 31% ~ 50% vii. Less than 30% ***Colors*** viii. Black and white ix. Others (specify) Image concept x. Positive outcome focused xi. Negative outcome focused Others – Congruency – whether the image is congruent with the text warnings
GHWs disclosure formats - Texts	***Location*** xii. All sides of a package xiii. Both the front and back xiv. On principal display areas (top, bottom) xv. Package inserts xvi. Others ***Size*** xvii. Legible font size xviii. Not legible font size ***Color*** xix. Black and white xx. Others (specify) ***Language*** ***Message content*** xxi. Positive outcome focused xxii. Negative outcome focused ***Source attribution (specify)***
	***Others (specify)***
Intervention sample	Sample description (e.g. people aged 18–25, smokers)
**C. Outcome Evaluation**	
Outcome measure - Benefits of quitting/Quit intentions	Pre-post changes across different groups
Outcome measure - Perceived risk of smoking	Pre-post changes across different groups

Trained coders (T.S., P.S.) extracted data from each record according to the published details contained in the publication. All of the records were cross checked by at least two independent coders to ensure consistency of data extraction.

### Quality assessment

To assess the risk of bias in included studies we applied either the Risk Of Bias In Non-Randomized Studies - of Interventions tool (ROBINS-I) [30] or Risk of Bias tool 2 (RoB2) [31] to each study selected into the systematic review. The ROBINS-I tool was used to evaluate the risk of bias in the results of non-randomized studies of interventions (NRSI) that compare the health effects of two or more interventions. This tool is highly applicable to the evaluation of GHW label studies because the types of NRSIs that can be evaluated using this tool are quantitative studies estimating the effectiveness (harm or benefit) of an intervention, which did not use randomization to allocate units (individuals or clusters of individuals) to comparison groups. This description applies to the majority of the studies selected into our review. The designs include studies where allocation occurs during the course of usual treatment decisions or peoples’ choices. In our systematic search results, the studies were of NRSI designs and nine papers were RCTs. For those RCTs, a Cochrane developed quality assessment framework – Risk of Bias Ver. 2 (RoB2) was used. RoB2 is a tool used to assess the risk of bias of the intervention effect between two intervention groups (the experimental and comparator group) [23]. This tool is applicable to the evaluation of quantitative GHW label studies ascertaining the effect of adhering to a controlled intervention (i.e. pictorial GHW, text only GHW or no GHW) where individuals were randomly assigned to intervention or comparator groups.

Each study was carefully examined and coded considering all the ways in which it might be put at risk of bias with close reference to the guidelines. Two independent reviewers (T.S., P.S.) coded each study and coding was compared. Any discrepancies were discussed and resolved with the help of a third and fourth reviewer (B.P., K.K.).

## Results

**Fig. 1:**
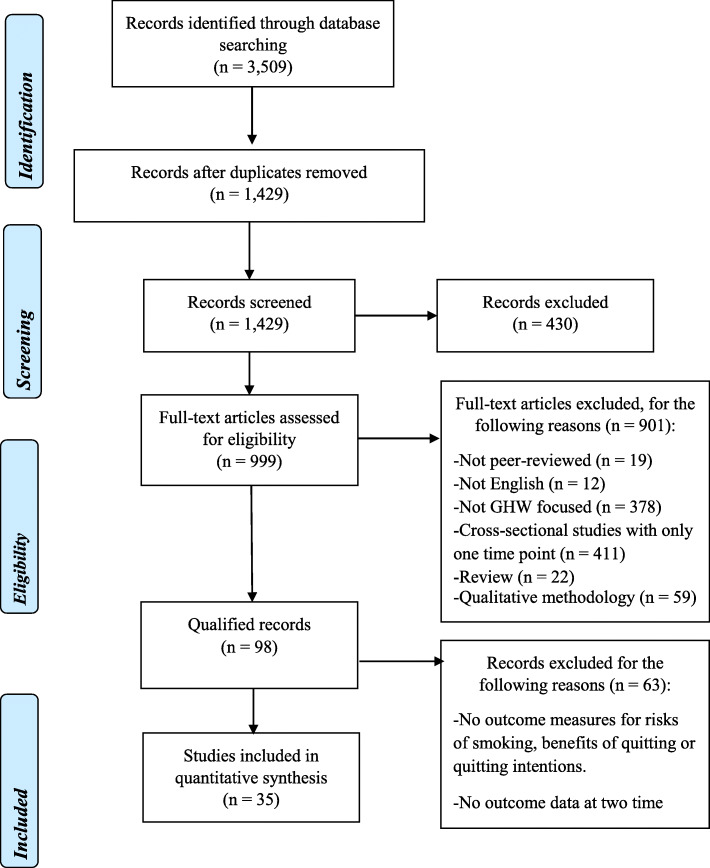
PRISMA Flow Diagram

After removing duplicates and ineligible articles, 35 studies met the inclusion criteria, resulting in the sample of qualified records. The PRISMA flow diagram summarizing the exclusion and inclusion process is shown in Fig. 1.

### Study Demographics

Overall, 40% of the studies were conducted across multiple countries (*n* = 14) such as Australia, Canada and Mexico [3, 32, 33], while 60% of interventions were conducted in one country (*n* = 21). Out of the 35 qualified studies, 63% of interventions were conducted in developed countries (*n* = 22), 29% in countries in development (*n* = 10), and 8% in both types of populations (*n* = 3). Frequently, data across a range of countries were reported in a single study, therefore, the quantity of counties mentioned is higher than the total of 35 studies. Developed countries evaluated include Australia (*n* = 12) [32, 34], the United States (n = 12) [35, 36], Canada (*n* = 11) [3, 33], Europe (*n* = 9) (five in the UK [1, 35], three in Germany [37–39], one in France and the Netherlands [38], and one in Italy [40]. Interventions conducted in countries in development (*n* = 10) included Asia (*n* = 6) (five in Malaysia [5, 41], three in Thailand [42, 43], one in China [5], one in India [4] and one in Vietnam [44], Latin America (*n* = 5) (four in Mexico [45], one in Uruguay [46] and one in Africa (in Mauritius [47]).

Regarding types of tobacco product packages that were tested, 86% of interventions were conducted on generic cigarette packs (*n* = 30), three were conducted on RYO cigarette packs [41, 43, 48], one was conducted on smokeless tobacco [4] and one was conducted on moist snuff, snus, and e-cigarettes [49]. The intervention group sample sizes varied from 44 [39] to 5991 [4]. Most of the studies were focused on smokers.

Over 70% of interventions were non-randomized designs (*n* = 26) followed by 26% of randomized interventions (*n* = 9) [39, 50]. Table 3 summaries the study demographics as well as key findings of included papers.

**Table 3:** Study characteristics

No.	Author(s), publication year	Location	Product	Study design	Sample size	Key findings
1	Anshari et al., 2018	Australia, Canada, Mexico	Cigarette packs	Longitudinal	AU: 1671	Over time, pictorial GHWs responses significantly changed in terms of increased noticing pictorial GHWs in Canada and Mexico, increased negative affect in Australia and decreased negative affect in Mexico.
					CA: 2357	
					MX: 2537	
2	Borland et al., 2009	Australia, Canada, UK, US	Cigarette packs	Longitudinal	AU: 4111	AU: all four indicators of impact increased following the introduction of GHW. Findings show partial wear-out of both graphic and text-only warnings, but the Canadian warnings have more sustained effects than UK ones.
					UK: 4273	
					CA: 4305	
3	Brewer et al., 2016	US	Cigarette packs	RCT	1071	Smokers who had pictorial GHWs were more likely than those with text-only GHWs to attempt to quit smoking during trial. Pictorial GHWs increased forgoing, intentions to quit, negative emotional reactions, thinking about the harms, and conversations about quitting.
4	Brewer et al., 2019	US	Cigarette packs	RCT	2149	Pictorial GHWs increased attention to, reactions to, and social interactions about warnings. However, pictorial GHWs changed almost no belief or attitude measures. Mediators of the impact of pictorial GHWs included harms of smoking and intentions to quit.
5	Cho et al., 2018	Australia, Canada, Mexico, US	Cigarette packs	Longitudinal	AU: 1036	Perceived risks significantly increased over time (AU & CA), where new more prominent GHWs included diseases that had not been described on prior GHWs. In MX, where pictures were changed but the diseases they described did not, perceived risks also increased.
					CA: 1190	
					MX: 1166	
6	Durkin et al., 2015	Australia	Cigarette packs and roll-your-own (RYO) packs	Longitudinal	*N* (weighted) = 5441	Plain Packaging (PP) early transition respondents showed significantly greater increases in stopping themselves from smoking and quit attempts. PP late transition respondents showed greater increases in intentions to quit and pack concealment. PP first year respondents showed higher levels of pack concealment, more premature stubbing and higher quit attempts.
7	Elton-Marshall et al., 2015	China, Malaysia	Cigarette packs	Longitudinal	2883	Significant changes prior to the new GHW introduction in noticing and reading GHWs. Compared to Malaysia, text-only GHWs in China led to a significant change in only two of six key indicators of GHW effectiveness: forgoing and reading the GHWs. The change to pictorial GHWs in Malaysia led to significant increases in five of six indicators (noticing, reading, forgoing, avoiding, thinking about quitting).
8	Fathelrahman et al., 2010	Malaysia	Cigarette packs	RCT	70	Exposure to pictorial GHWs increased awareness of risks, behavioral response and quitting intention. Interest in quitting increased significantly more in those exposed to pictorial GHWs.
9	Fathelrahman et al., 2013	Malaysia, Thailand	Cigarette packs	Longitudinal	Pre GHW: 1018	Multivariate predictors of “interest in quitting” were comparable across countries, but predictors of quit attempts varied. In both countries, cognitive reactions, forgoing and baseline knowledge were positively associated with interest in quitting at that wave. Thailand only: cognitive reactions, forgoing a cigarette” and interest in quitting were positively associated with quit attempts over the following inter-wave interval.
					Post GHW: 803	
10	Glock & Kneer, 2009	Germany	Cigarette packs	RCT	60	There was no major effect from the intervention condition, and after being confronted with warning labels, smokers decreased their perceived smoking-related risk.
11	Gravely et al., 2016a	Uruguay	Cigarette packs	Longitudinal	Wave 2: 1294	All indicators of GHW effectiveness increased significantly, including salience, thinking about risks, thinking about quitting, avoiding looking, and stopping from having a cigarette ‘many times’.
					Wave 3: 452	
12	Gravely et al., 2016b	India	Smokeless tobacco	Longitudinal	Scorpion GHW: 5991	GHW label change in India from symbolic (scorpion) to pictorial GHWs did not result in significant increases on any of the GHW outcome indicators.
					New pictorial GHW: 4634	
13	Green et al., 2014	Mauritius	Cigarette packs	Longitudinal	Pre (w1): 598	All indicators of warning effectiveness (salience, cognitive, and behavioral reactions) and the Label Impact Index (weighted combination of 4 indicators) increased significantly between Waves 1 and 2. However, between Waves 2 and 3, there was a significant decline in the proportion of smokers who reported “avoiding looking” at labels.
					Post 12 months (w2): 555	
14	Green et al., 2019	Canada	Cigarette packs	Longitudinal	5863	Adding messages to GHWs significantly increased awareness that smoking causes blindness and bladder cancer. Adding the warning that nicotine causes addiction did not significantly impact smokers’ awareness. Removing messages was shown to decrease awareness that cigarette smoke contains carbon monoxide and smoking causes impotence.
15	Hall et al., 2018	US	Cigarette packs	RCT	1071	Pictorial GHWs increased negative affect, message reactance and quit intentions, but not perceived risk. Negative affect mediated the impact of pictorial warnings on quit intentions.
16	Hitchman et al., 2014	Canada, US	Cigarette packs	Longitudinal	CA: 5309	The effectiveness of both pictorial GHWs (CA) and text-only GHWs (US) warnings declined significantly over time. Pictorial GHWs showed greater declines in effectiveness than the text-only warnings. Despite the greater decline in pictorial GHWs, they were significantly more effective than the text-only GHWs throughout the study.
17	Kasza et al., 2017	Australia, Canada, UK, US	Cigarette packs	Longitudinal	CA: 4884	Between 2002 and 2015, smokers’ concern for personal health was the most frequently endorsed reason for thinking about quitting in the UK, Canada, the US and Australia, and across all reasons to quit smoking, concern for personal health had the strongest association with making a quit attempt at follow-up wave.
					AU: 4482	
18	Kennedy et al., 2012	Australia, Canada, UK, US	Cigarette packs	Longitudinal	AU: 3151	After the introduction of the blindness warning, Australian smokers were more likely than before the blindness warning to report that they know that smoking causes blindness. In Australia, smokers aged over 55 years were less likely than those aged 18 to 24 to report that smoking causes blindness.
19	Li et al., 2015	Australia, Canada, UK	Cigarette packs	Cohort	AU (t1): 1801	The impact of warnings declined over time in all three countries. Having two rotating sets of warnings does not appear to reduce wear-out over a single set of warnings. Warning size may be more important than warning type in preventing wear-out, although both probably contribute interactively.
					AU (t2): 1104	
20	Li et al., 2016	Malaysia, Thailand	Cigarette packs and RYO packs	Longitudinal	TH (w3): 2465	The main outcome was subsequent quit attempts. Following the implementation of GHWLs in Malaysia, reactions increased, in some cases to levels similar to the larger Thai warnings, but declined over time. In Thailand, reactions increased following implementation, with no decline for several years, and no clear effect of the small increase in warning size. Reactions, mainly cognitive responses, were consistently predictive of quit attempts in Thailand, but this was only consistently so in Malaysia after the change to GHWLs.
					Th (w5): 2132	
					MY (w2): 1640	
					MY (w4): 2045	
					MY (w6): 2000	
21	Mannocci et al., 2019	Italy	Cigarette packs	Longitudinal	Pre: 788	Significant increases of knowledge of health risk after pictorial GHWs introduction in a short period (8–18 months). The awareness about gangrene, blindness, premature labour and erectile dysfunction registered the higher increase before and after law implementation.
					Post: 455	
22	Mays et al., 2014	US	Cigarette packs	RCT	740	Gain-framed warnings generated significantly greater motivation to quit among smokers with high perceived risks compared with smokers with low perceived risks. Among smokers with high perceived risks, gain-framed messages were superior to loss-framed messages.
23	McQueen et al., 2015	US	Cigarette packs	Longitudinal	202	Participants reported low avoidance and consistent use of the stickers. Smokers consistently paid more attention to graphic than text-only labels. Only 5 of the 9 GHWs were significantly associated with greater thoughts of health risks. Thinking about quitting and stopping smoking did not differ by label.
24	Nagelhout et al., 2016	France, Germany, Netherlands	Cigarette packs	Longitudinal	UK: 1643	Salience decreased between the surveys in France and showed a non-significant increase in the UK, cognitive responses increased in the UK and decreased in France, forgoing cigarettes increased in the UK and decreased in France, and avoiding warnings increased in France and the UK.
					FR: 1540	
25	Ngan et al., 2016	Vietnam	Cigarette packs	Longitudinal	Wave 1: 1462	Two years after implementation, salience of the pictorial GHWs was higher than one year after implementation. The proportion of respondents who tried to avoid noting pictorial GHWs decreased from 35% in wave 1 to 23% in wave 2. However, avoidance increased 1.5 times the odds of presenting quit intention compared to those respondents who did not try to avoid looking/thinking about the pictorial GHWs
					Wave 2: 1509	
26	Nicholson et al., 2017	Australia	Cigarette packs	Cohort	642	Forgoing increased significantly only for those first surveyed prior to the introduction of plain packaging (PP); however, there were no significant interactions between forgoing and the introduction of new and enlarged graphic warning labels on PP in any model.
27	Osman et al., 2016	Mexico	Cigarette packs	Longitudinal	1340	All GHW responses increased over time, except putting off smoking.
28	Parada et al., 2017	US	Cigarette packs	RCT	Intervention: 1071	Smokers in the intervention (pictorial GHWs) group thought more about the warning message and harms of smoking, reported higher levels of fear due to warnings, experienced more negative affect, expressed more intention to quit, and forewent smoking cigarettes more than participants in the control group.
					Control: 1078	
29	Partos et al., 2013	Australia, Canada, UK, US	Cigarette packs	Longitudinal	AU: 576	Reporting that GHWs make quitting over time ‘a lot’ more likely (compared with ‘not at all’ likely) was associated with a lower likelihood of relapse 1 year later and this effect remained robust across all models tested, increasing in some. Reporting that GHWs make you more likely to remain smoking free was strongly correlated with reporting that GHWs make you think about health risks.
					CA: 478	
					UK: 512	
30	Popova & Ling, 2014	Canada	Moist snuff, snus, and e-cigarettes	RCT	76	Pictorial GHWs increased perceived harm of moist snuff and e-cigarettes. Current warning label and pictorial GHW significantly lowered positive attitudes towards e-cigarettes.
31	Schneider et al., 2012	Germany	Cigarette packs	RCT	44	Pictorial GHWs were associated with a significantly higher motivation to quit. A pictorial GHW was also associated with higher fear intensity. The effect of warnings appears to be independent of nicotine dependence and self-affirmation.
32	Swayampakala et al., 2014	Australia, Canada, Mexico	Cigarette packs	Longitudinal	AU: 1001	Smokers in countries with GHWs describing specific health risks had greater awareness and knowledge of those specific health risks (with only few exceptions) compared to smokers in countries that do not include the same GHWs health risks (e.g., risk of blindness in Australia, but not Mexico).
					CA: 1001	
					MX: 1000	
33	White et al., 2008	Australia	Cigarette packs	Longitudinal	2432	Attention to and processing of warning labels increased from T1 to T2. Smokers considered quitting more at follow-up (T2).
34	Yong et al., 2013	Thailand, Malaysia	Cigarette packs and RYO packs	Longitudinal	TH (w1): 3067	After GHW change smokers’ awareness, cognitive, and behavioral reactions increased, with cognitive and behavioral effects sustained at follow-up (Thailand). Compared to smokers who smoke generic cigarettes, smokers of RYO reported lower salience but greater cognitive reactions to the new pictorial GHWs.
					TH (w2): 1986	
35	Yong et al., 2016	Australia	Cigarette packs	Longitudinal	Pre: 1104	Attentional orientation towards GHWs and reported frequency of noticing warnings increased significantly after the policy change, but not more reading. Smokers also thought more about the harms of smoking and avoided the GHWs more after the policy change, but frequency of forgoing cigarettes did not change.
					Post: 1093	

### Quality assessment

Two tools were used to assess study quality. These are presented in turn. The ROBINS-I tool was applied to 26 of the 35 included studies due to the NRSI nature of the studies. The majority (96.2%) of the 26 studies were found to be at moderate risk of bias in at least one of the domains. The moderate overall bias was attributed mostly to ‘confounding’ variables. Confounding was predominately due to introduction of enhanced pictorial health warnings [3, 35], other marketing campaigns and smoking policies (e.g. price increases) during the course of the longitudinal study [34, 48], or due to variance in smoking quantity amongst participants [32, 38, 51]. One study was found to be serious in at least one domain [1]. Partos, Borland [1] was rated as serious in the ‘selection of participants’ domain because selection into the study was based on self-report of quitting smoking and the main outcome measures were quit-related with no note on whether selection bias was taken into account. Over half of the studies (18 out of 26 studies) were at low risk of bias due to missing data. Many studies had almost complete outcome data or used suitable data analysis methods to minimise the bias from missing data. Table 4 summarizes risk of bias ratings for individual studies.

**Table 4:** Quality assessment

No.	Author(s), publication year	Risk of bias due to confounding	Risk of bias in selection of participants	Risk of bias in classification of interventions	Risk of bias due to deviations from intended intervention	Risk of bias due to missing data	Risk of bias in measurement of outcomes	Risk of bias in selection of the reported result	Overall
1	Anshari et al., 2018	Moderate	Moderate	Moderate	Low	Low	Moderate	Low	**Moderate**
2	Borland et al., 2009	Moderate	Low	Moderate	Low	Low	Moderate	Low	**Moderate**
5	Cho et al., 2018	Moderate	Moderate	Moderate	Low	Low	Moderate	Low	**Moderate**
6	Durkin et al., 2015	Moderate	Low	Low	Low	Moderate	Moderate	Low	**Moderate**
7	Elton-Marshall et al., 2015	Moderate	Low	Low	Low	Moderate	Low	Low	**Moderate**
9	Fathelrahman et al., 2013	Moderate	Low	Low	Low	Moderate	Low	Low	**Moderate**
11	Gravely et al., 2016a	Moderate	Low	Low	Low	Moderate	Moderate	Low	**Moderate**
12	Gravely et al., 2016b	Moderate	Low	Low	Low	Low	Moderate	Low	**Moderate**
13	Green et al., 2014	Moderate	Low	Low	Low	Low	Moderate	Low	**Moderate**
14	Green et al., 2019	Moderate	Low	Moderate	Low	Low	Moderate	Low	**Moderate**
16	Hitchman et al., 2014	Moderate	Low	Low	Low	Low	Moderate	Low	**Moderate**
17	Kasza et al., 2017	Moderate	Low	Low	Low	Low	Low	Low	**Moderate**
18	Kennedy et al., 2012	Moderate	Low	Low	Low	Low	Moderate	Low	**Moderate**
19	Li et al., 2015	Moderate	Moderate	Low	Low	NI	Moderate	Low	**Moderate**
20	Lin et al., 2016	Moderate	Moderate	Low	Low	Low	Moderate	Low	**Moderate**
21	Mannocci et al., 2019	Moderate	Moderate	Moderate	Low	NI	Moderate	Low	**Moderate**
22	Mays et al., 2014	Moderate	Low	Low	Low	Low	Moderate	Low	**Moderate**
24	Nagelhout et al., 2016	Moderate	Moderate	Low	Low	Low	Moderate	Low	**Moderate**
25	Ngan et al., 2016	Moderate	Low	Moderate	Low	Low	Moderate	Low	**Moderate**
26	Nicholson et al., 2017	Moderate	Moderate	Moderate	Low	Low	Moderate	Low	**Moderate**
27	Osman et al., 2016	Moderate	Low	Low	Low	Low	Low	Low	**Moderate**
29	Partos et al., 2013	Moderate	Serious	Low	Low	Low	Moderate	Low	**Serious**
32	Swayampakala et al., 2014	Moderate	Low	Low	Low	Low	Moderate	Low	**Moderate**
33	White et al., 2008	Moderate	Low	Low	Low	Moderate	Moderate	Low	**Moderate**
34	Yong et al., 2013	Moderate	Low	Low	Low	Moderate	Moderate	Low	**Moderate**
35	Yong et al., 2016	Moderate	Low	Moderate	Low	Low	Moderate	Low	**Moderate**

RoB2

The RoB2 tool was applied to accurately address bias in nine studies that were identified as RCTs. Six studies were judged to be at a low risk of bias among all domains [37, 39, 49, 50, 52, 53]. Two studies were judged to have some concerns in at least one domain [36, 54]. Parada, Hall [36] was low in all domains except for bias due to missing outcome data. One paper [36] stated “Proportions of participants who completed the follow-up surveys at each of the follow up weeks included 86% at week 1, 82% at week 2, 81% at week 3 and 84% at week 4” (p 878). No information regarding how missing data was handled or if the missing data biased the final result was provided lowering assessment scores. Fathelrahman, Omar [54] was rated as some concerns in effect of assignment to interventions but was low in all other domains. There was unclear information on whether participants were blinded to the intervention because “In both groups, participants were given the packs all at the same time and instructed to take a few minutes to examine them” (p. 4091). In addition, research staff were aware of the intervention that was assigned to participants as they assigned the cigarette packaging.

One study was judged to be a high risk of bias overall as a result of a high risk from the randomization process [55]. There was no description of the randomization process. Furthermore, there were substantial differences between intervention group sizes, compared with the intended allocation ratio. Group differences were not examined “because of small cell sizes, we did not examine statistical differences in socio-demo- graphics by study condition” (p. 787). For all other domains, McQueen, Kreuter [55] was judged to be at low risk of bias. Table 5 presents details on RoB2 assessment applied to randomized controlled trials included in this review.

**Table 5:** Quality assessment

No.	Author(s), publication year	Risk of bias arising from the randomization process	Risk of bias due to deviations from the intended interventions (effect of assignment to intervention)	Risk of bias due to missing outcomes data	Risk of bias in measurement of the outcome	Risk of bias in selection of the reported result	Overall risk of bias
3	Brewer et al., 2016	Low	Low	Low	Low	Low	**Low**
4	Brewer et al., 2019	Low	Low	Low	Low	Low	**Low**
8	Fathelrahman et al., 2010	Low	Some concerns	Low	Low	Low	**Some concerns**
10	Glock & Kneer, 2009	Low	Low	Low	Low	Low	**Low**
15	Hall et al., 2018	Low	Low	Low	Low	Low	**Low**
23	McQueen et al., 2015	High	Low	Low	Low	Low	**High**
28	Parada et al., 2017	Low	Low	Some concerns	Low	Low	**Some concerns**
30	Popova & Ling, 2014	Low	Low	Low	Low	Low	**Low**
31	Schneider et al., 2012	Low	Low	Low	Low	Low	**Low**

RoB2

### GHW formats

GHWs vary in formatting according to the size of image, placement on the package, message framing, congruence, colour, text size, message attribution (source), and the inclusion of Quitline information. The included studies described warning formats for images, with sizes ranging from 30 to 90% coverage of cigarette packaging. Formats in front of the cigarette package varied from 30% (*n* = 13) [35, 56], 50% (*n* = 15) [41, 50] to 75% (*n* = 8) [32, 34], and included other sizes (*n* = 10) between 40% [5] and 90% [33]. Formats on the back of the cigarette package varied from 30% (*n* = 1) [37], 50% (*n* = 14) [41, 50] to 75% (*n* = 4) [33, 57]. Once again other sizes (*n* = 7) such as 40% [38], 80% [46], 90% (*n* = 13) [34, 48] and 100% (*n* = 3) were described [3, 32, 33].

In regard to the location of the images on the cigarette pack, 77% of the studies (*n* = 27) evaluated front and back image formats. Twelve studies evaluated other location formats including only the front image (*n* = 5) [39, 54], front, back & sides (*n* = 1) [32], front, back & package inserts (*n* = 4) [3, 33, 47, 57], and no location on the pack (GHW images shown on a screen) (*n* = 2) [37, 49].

In regard to message framing, 91% of the studies (*n* = 32) evaluated negatively framed message formats. Negative framing refers to fear-based images, for instance, a graphic photo of a severe disease (e.g. mouth cancer) in Australian GHWs [35]. Nine studies evaluated other types of message framing including symbolic messages (*n* = 3), for example, an image of a scorpion to communicate danger for Indian GHWs on smokeless tobacco [4], or an empty cradle paired with the message ‘tobacco hurts everyone’ in Canadian GHWs [58], and mixed (*n* = 6) framing where negative and symbolic GHW formats in the same groups [3, 32, 33, 47, 57, 59] were evident.

GHW formats where the message was presented in images and text communicating a consistent, aligned message is a key factor for GHW effectiveness. One study evaluated an incongruent message format, notably a scorpion GHW that was not congruent with the message ‘tobacco kills’ that was implemented when the GHW was changed in India [4]. Likewise, 94% of the studies (*n* = 33) evaluated color GHW image formats. Only two studies evaluated black and white formats from RYO cigarette packs in Thailand [41, 43].

In regards to text formats that accompany GHWs, text size varies from approximately 10% of the package, for instance in Malaysia [5] and the UK [1], to 100% of the back of the package in the case of Mexico [3]. Fifty-four percent of studies evaluated formats that displayed a text source of attribution (*n* = 19). For instance, the ‘Health Authority Warning’ source that appeared in 2006–2012 Australian GHWs (*n* = 9) [34, 35], Health Canada (*n* = 8) [32, 47], and US Department of Health and Human Services (US HHS) (*n* = 5) [55, 60]. 46% of studies evaluated formats that did not display any source of attribution (*n* = 16) [42, 46]. Forty-three percent of studies tested formats that displayed a quit line number (*n* = 15), for instance, Australian GHW formats before and after plain packaging policy [34]. Finally, 37% of studies tested formats that did not display any quit line number (*n* = 13), such as Uruguay [46], and 17% of studies tested both formats (*n* = 6). Table 6 summarizes the formatting features of included studies.

**Table 6:** Formatting of GHWs

No.	Author(s), publication year	Group	Graphic: Size (front) %	Graphic: Size (back) %	Graphic: Location	Graphic: Image concept^**1**^	Graphic: congruency^**2**^	Graphic: Colour	Text: Size %	Text: Location	Text: Source attribution	Text only GHW	Quitline
1	Anshari et al., 2018	Australia	75	90	Front & back	Negative	Congruent	Colour	25	Front & back	No source	No	Yes
		Canada	75	75	Front, back & sides	Mixed	Congruent	Colour	37	Front, back & sides	Health Canada	No	Yes
		Mexico	30	100	Front & back	Negative	Congruent	Colour	100	Back	No source	No	Yes
2	Borland et al., 2009	Australia (Wave2)	NA	NA	NA	NA	NA	NA	NA	NA	NA	Yes	No
		Australia (Wave5)	30	90	Front & back	Negative	Congruent	Colour	15	Front & back	Health authority warning	No	Yes
		UK	NA	NA	NA	NA	NA	NA	NA	NA	NA	Yes	No
		Canada	50	50	Front & back	Negative	Congruent	Colour	20	Front & back	Health Canada	No	No
3	Brewer et al., 2016	Pictorial (Pre & Post)	50	50	Front & back	Negative	Congruent	Colour	12.5	Front & back	US HHS	No	No
4	Brewer et al., 2019	Complete sample	50	50	Front & back	Negative	Congruent	Colour	15	Front & back	US HHS	No	No
5	Cho et al., 2018	Australia	75	90	Front & back	Negative	Congruent	Colour	25	Front & back	No source	No	Yes
		Canada	75	75	Front, back & package inserts	Mixed	Congruent	Colour	37	Front, back & package inserts	Health Canada	No	Yes
		Mexico	30	100	Front & back	Negative	Congruent	Colour	100	Back	No source	No	Yes
6	Durkin et al., 2015	Pre PP	30	90	Front & back	Negative	Congruent	Colour	15	Front & back	Health authority warning	No	Yes
		Late transition & Post PP	75	90	Front & back	Negative	Congruent	Colour	25	Front & back	No source	No	Yes
7	Elton-Marshall et al., 2015	Malaysia (Pre)	NA	NA	NA	NA	NA	NA	NA	NA	NA	Yes	No
		Malaysia (Post)	40	60	Front & back	Negative	NA	Colour	10	Front	No source	No	Yes
8	Fathelrahman et al., 2010	Complete sample	60	NA	Front	Negative	Congruent	Colour	40	Front	No source	No	Yes
9	Fathelrahman et al., 2013	Pre GHW	NA	NA	NA	NA	NA	NA	NA	NA	NA	Yes	No
		Post GHW	50	50	Front & back	Negative	Congruent	Colour	10	Front	No source	No	No
10	Glock & Kneer, 2009	Complete sample	30	30	No location on pack (screen)	Negative	Congruent	Colour	NA	NA	NA	No	No
11	Gravely et al., 2016a	Wave 2	50	50	Front & back	Symbolic	NA	Colour	30	Front	No source	No	No
		Wave 3	80	80	Front & back	Negative	Congruent	Colour	30	Front & back	No source	No	No
12	Gravely et al., 2016b	Scorpion GHW	40	NA	Front	Symbolic	Incongruent	NA	NA	Front	NA	No	NA
		New pictorial GHW	40	NA	Front	Negative	Congruent	Colour	NA	Front	NA	No	NA
13	Green et al., 2014	Pre pictorial GHW (Wave1)	NA	NA	NA	NA	NA	NA	30	NA	NA	Yes	NA
		Post 12 months (Wave2)	60	70	Front & back	Mixed	Congruent	Colour	65 (side)	Front & back	No source	No	No
14	Green et al., 2019	Complete sample	75	90	Front, back & package inserts	Mixed	Congruent	Colour	37	Front, back & package inserts	Health Canada	No	Yes
15	Hall et al., 2018	Complete sample	50	50	Front & back	Negative	Congruent	Colour	15	Front & back	US HHS	No	YES
16	Hitchman et al., 2014	Canada	50	50	Front & back	Symbolic	Congruent	Colour	25	Front & back	Health Canada	No	No
17	Kasza et al., 2017	Canada (Wave1)	50	50	Front & back	Negative	Congruent	Colour	25	Front & back	Health Canada	No	No
		Canada (Wave9)	75	75	Front, back & package inserts	Mixed	Congruent	Colour	37	Front, back & package inserts	Health Canada	No	Yes
		Australia (Wave1)	NA	NA	NA	NA	NA	NA	NA	NA	NA	Yes	No
		Australia (Wave9)	75	90	Front & back	Negative	Congruent	Colour	25	Front & back	No source	No	Yes
18	Kennedy et al., 2012	Australia (Waves 1–7)	30	90	Front & back	Negative	Congruent	Colour	15	Front & back	Health authority warning	No	Yes
19	Li et al., 2015	Australia	30	90	Front & back	Negative	Congruent	Colour	15	Front & back	Health authority warning	No	Yes
20	Li et al., 2016	Thailand (Wave3)	50	50	Front & back	Negative	Congruent	Black & white	10	Front & back	No source	No	No
		Thailand (Wave5)	55	55	Front & back	Negative	Congruent	black & white	10	Front & back	No source	No	Yes
		Malaysia (Wave2)	NA	NA	NA	NA	NA	NA	70	Sides	NA	Yes	No
		Malaysia (Wave4 and6)	40	60	Front & back	Negative	Congruent	Colour	10	Front & back	No source	No	Yes
21	Mannocci et al., 2019	Italy (Post)	65	65	Front & back	Negative	Congruent	Colour	50	Sides	No source	No	No
22	Mays et al., 2014	Intervention	50	50	Front & back	Negative	NA	Colour	NA	Front & back	US HHS	No	Yes
23	McQueen et al., 2015	Graphic condition	50	NA	Front	Negative	Congruent	Colour	NA	Front	US HHS	No	Yes
24	Nagelhout et al., 2016	UK (Pre)	NA	NA	NA	NA	NA	NA	30 (front), 40 (back)	3	NA	Yes	NA
		UK (Post)	43	53	Front & back	Negative	Congruent	Colour	10	Front & back	No source	No	No (only 1/14 has Quitline)
		FR (Pre)	NA	NA	NA	NA	NA	NA	30 (front), 40 (back)	3	NA	Yes	NA
		FR (Post)	0	40	Front & back	Negative	Congruent	Colour	20	Front	No source	No	Yes
25	Ngan et al., 2016	Wave 1 & 2	50	50	Front & back	Negative	Congruent	Colour	12	Front & back	No source	No	No
26	Nicholson et al., 2017	Pre PP	30	90	Front & back	Negative	Congruent	Colour	15	Front & back	Health authority warning	No	Yes
		Post PP	75	90	Front & back	Negative	Congruent	Colour	25	Front & back	No source	No	Yes
27	Osman et al., 2016	Complete sample	30	0	Front	Negative	Congruent	Colour	100	Back	No source	No	Yes
28	Parada et al., 2017	Pictorial GHW (Waves 1–4)	50	50	Front & back	Negative	Congruent	Colour	NA	Front & back	No source	No	No
29	Partos et al., 2013	AU	30	90	Front & back	Negative	Congruent	Colour	15	Front & back	Health authority warning	No	Yes
		CA	50	50	Front & back	Negative	Congruent	Colour	25	Front & back	Health Canada	No	No
		UK	30	40	Front & back	Negative	Congruent	Colour	10	Front	No source	No	No
30	Popova & Ling, 2017		NA	NA	No location on pack (screen)	Negative	Congruent	Colour	NA	No location on pack (screen)	No source	No	No
31	Schneider et al., 2012	Pictorial GHW	45	0	Front	Negative	Congruent	Colour	20	Front	No source	No	No
32	Swayampakala et al., 2014	AU (Pre)	30	90	Front & back	Negative	Congruent	Colour	15	Front & back	Health authority warning	No	Yes
		AU (Post)	90	90	Front & back	Negative	Congruent	Colour	30	Front & back	No source	No	Yes
		CA (Pre)	75	75	Front & back	Mixed	Congruent	Colour	25	Front & back	Health Canada	No	Yes
		CA (Post)	75	75	Front, back & package inserts	Negative	Congruent	Colour	25	Front, back & package inserts	Health Canada	No	Yes
		MX (Pre-Post)	30	100	Front & back	Negative	Congruent	Colour	100	Back	No source	No	Yes
33	White et al., 2008	AU 2006 GHW	30	90	Front & back	Negative	Congruent	Colour	15	Front & back	Health authority warning	No	Yes
34	Yong et al., 2013	TH (Wave1)	NA	NA	NA	NA	NA	NA	33	Front & back	NA	Yes	NA
		TH (Wave 3)	50	50	Front & back	Negative	Congruent	black & white	12	Front & back	No source	No	No
35	Yong et al., 2016	Pre PP	30	90	Front & back	Negative	Congruent	Colour	15	Front & back	Health authority warning	No	Yes
		Post PP	75	90	Front & back	Negative	Congruent	Colour	25	Front & back	No source	No	Yes

^1^Positive image concept refers to graphics portraying the benefits of quitting smoking while negative images show the harmful effects of smoking.

^2^Congruency refers to GHW formats where the message is presented in images and text communicating a consistent, aligned message

### Perceived harm outcomes

Thirty-four studies measured the impact of changes to GHWs on perceived risk using various measures across studies and countries. To be noted that the measurement of perceived risks is substantially inconsistent across included studies. In eight studies, a single measure of “To what extent, if at all, do the health warning labels make you think about the health risks of smoking” (or slight variation of wording) was used to measure the perceived risk of smoking [1, 5, 41, 45, 46, 54, 55, 58, 59]. Seven studies used combined “cognitive response” measures [4, 34, 35, 38, 42, 43, 61]. See the Table 7 for the detailed measures. The perceived risk measure of “extent to which the warnings made the respondent think about the health risks of smoking” was often combined with the “if the health warnings made them more likely to quit” to cognitive reactions. Other commonly used measures were perceived likelihood of harm which were used in three studies [50, 52, 53] and identifying that smoking causes tobacco related diseases was used in six studies [3, 33, 37, 47, 56, 62]. A brief summary of the findings is presented in Table 7.

**Table 7:** Perceived harm outcomes

No.	Author(s), publication year	Group	Perceived risk		OR or Beta	Measures
			Pre	Post		
1	Anshari et al., 2018	Australia	0.22 (0.03 to 0.40)	0.36 (0.09 to 0.63)	NA	Negative affect: “How much does this warning make you feel worried about the health risks of smoking?”
		Canada	0.06 (−0.08 to 0.20)	0.03 (−0.23 to 0.28)	NA	
		Mexico	0.00 (−0.14 to 0.15)	−0.25 (−0.47 to 0.02)	NA	
2	Borland et al., 2009*	Australia	1.68	2.04	NA	Cognitive responses combined two questions: “Extent to which the warnings both made the respondent think about the health risks of smoking” and “made them more likely to quit smoking”
		UK	1.95	1.81	NA	
		Canada	1.93	1.84	NA	
3	Brewer et al., 2016	NA	3.3 (0.9)	3.4 (0.9)	NA	Combined three perceived harm questions: “What is the chance that you will one day get cancer if you continue to smoke cigarettes?”, “What is the chance that you will one day get heart disease if you continue to smoke cigarettes?” and “What is the chance that you will one day get a permanent breathing problem if you continue to smoke cigarettes?”
4	Brewer et al., 2019	NA	0.04 (−0.04, 0.13)	0.03 (−0.05, 0.11)	NA	Perceived likelihood of harm from smoking combined 3 questions: “What is the chance that you will one day get cancer if you continue to smoke cigarettes?”, “What is the chance that you will one day get heart disease if you continue to smoke cigarettes?” and “What is the chance that you will one day get a permanent breathing problem if you continue to smoke cigarettes?”
5	Cho et al., 2018	Australia	1.14	1.22	NA	“Indicate which illnesses, if any, are caused by smoking cigarettes (emphysema, heart attacks, bladder cancer, blindness, impotence in male smokers, gangrene, hepatitis, and diseases that lead to amputation)” and ‘Their own chance of getting the disease in the future to the chance of a nonsmoker if they continue to smoke the amount that they currently do’
		Canada	1	1.22	NA	
		Mexico	1.25	1.26	NA	
7	Elton-Marshall et al., 2015	NA	6.90%	11.80%	NA	“To what extent, if at all, do the health warnings on cigarette packs make you more likely to think about the health risks (health danger) of smoking?”
8	Fathelrahman et al., 2010	NA	8 (11.6%)	20 (29.0%)	NA	“To what extent, if at all, do the health warnings on the cigarette pack designs make you more likely to quit smoking”
9	Fathelrahman et al., 2013	NA	3.6 (1.9)	3.8 (2.0)	NA	Cognitive reactions combined two measures: “thinking about health risk because of them (think-harm)” and thinking about quitting because of them (think-quit)”
10	Glock & Kneer, 2009	NA	5.50 (2.05)	4.67 (1.63)	NA	Pre health warning viewing: Six smoking-related and six non-smoking-related diseases were rated between 0 (no risk of developing disease) and 9 (highest risk of developing disease). Post health warning viewing: rated another 12 diseases under the same conditions
11	Gravely et al., 2016a	NA	31.5%	43.3%	OR: 1.66	“To what extent do the health warnings make you think about the dangers from smoking?”
12	Gravely et al., 2016b	NA	15.0 (95% CI 11.9; 18.8)	17.5 (95% CI 12.1; 24.6)	NA	Cognitive reactions two questions: “To what extent, if at all, do the warning labels on smokeless tobacco packages make you more likely to think about the health risks (health danger) of using it?” and “To what extent, if at all, do the warning labels on smokeless tobacco packages make you more likely to quit using it?”
13	Green et al., 2014	NA	24.50%	41.80%	OR: 2.47 (95% CI = 1.87–3.26)	“To what extent, if at all, do the warning labels make you think about the health risks of smoking”
14	Green et al., 2019	Blindness	14.70%	36.70%	NA	“based on what you know or believe, does smoking cause (stroke, impotence, bladder cancer and blindness)” **Note:** blindness, bladder cancer and addiction were chosen because they were the new messages added to health warning labels
		Bladder Cancer	26.80%	44.00%	NA	
		Addiction	90.50%	89.60%	NA	
15	Hall et al., 2018	NA	3.3 (0.9)	3.55 (0.63)	NA	Perceived likelihood of harm combined 3 questions: “What is the chance that you will one day get heart disease if you continue to smoke cigarettes?”, “What is the chance that you will one day get cancer if you continue to smoke cigarettes?” and “What is the chance that you will one day get a permanent breathing problem if you continue to smoke cigarettes?”
16	Hitchman et al., 2014*	NA	NA	NA	Log OR: −0.320 (×2 = 5.45)	“To what extent, if at all, do the warning labels make you think about the health risks of smoking”
17	Kasza et al., 2017	Canada	NA	NA	OR: 1 (CI 1.00 to 1.01)	Concern/ risk reasons: “concern for personal health”, “setting example for children” and “concern for health of others”
		Australia	NA	NA	OR: 1.01 (CI 1.00 to 1.01)	
18	Kennedy et al., 2012	Australia (Waves 1–7)	50.1	69.5	NA	‘I am going to read you a list of health effects and diseases that may or may not be caused by smoking cigarettes. Based on what you know or believe.’ This statement was followed by possible health effects, including, ‘does smoking cause blindness?’
		Australia (Wave 8)	69.5	57.5		
19	Li et al., 2015	NA	2.1	1.9	NA	Cognitive response combined 3 questions: “made them think about the health risks of smoking”, “made them more likely to quit smoking” and “if ‘warning labels on cigarette packages’ motivated them to think about quitting in the past 6 months”
20	Li et al., 2016	Thailand	0.49 (0.06)	0.61 (0.06)	NA	Cognitive response combined 2 questions: “made them think about the health risks of smoking” and “made them more likely to quit smoking”
		Malaysia (Waves 2–4)	0.07 (0.06)	1.01 (0.06)	NA	
		Malaysia (Waves 4–6)	1.01 (0.06)	0.47 (0.06)	NA	
21	Mannocci et al., 2019	NA	11.6 (2.5)	14.6 (1.8)	NA	“Identify tobacco related illnesses (from a list of 20 diseases)”
22	Mays et al., 2014	NA	2.2 (1.1)	3.5 (1.3)	NA	Perceptions of warnings “warnings convey risks”
23	McQueen et al., 2015	NA	146 (79%)	158 (86%)	NA	“Made them think about the health risks of smoking”
24	Nagelhout et al., 2016	UK	NA	NA	OR: 1.34	Cognitive responses combined 3 questions: “To what extent, if at all, do the warning labels make you think about the health risks of smoking?”, “To what extent, if at all, do the warning labels on cigarette packs make you more likely to quit smoking?” and “In the past 6 months, have warning labels on cigarette packages led you to think about quitting?”
		France	NA	NA	OR: 0.7	
25	Ngan et al., 2016	NA	12.7	18.8	NA	“Do you worry about the health consequences of smoking?”
26	Nicholson et al., 2017	NA	35	38	NA	“Very worried that smoking will damage your health in future”
27	Osman et al., 2016	NA	NA	NA	b = 0.23, SE = 0.03, *p* < .001	“To what extent, if at all, do the health warnings make you think about the health risks of smoking?”
28	Parada et al., 2017*	NA	3.2 (mean)	3.1	NA	two questions: “In the last week, how often did you think about the harm your smoking might be doing to you?” and “In the last week, how often did you think about the harm your smoking might be doing to other people?.”
29	Partos et al., 2013*	Australia (Waves 4–6)	1.89	2.42	NA	“To what extent, if at all, do the warning labels make you think about the health risks of smoking”
		Canada (Waves 2–6)	2.46	2.4	NA	
		UK (Waves 2–6)	2.49	2.07	NA	
30	Popova & Ling, 2014	NA	6.86	7.57	NA	Two questions: ‘In your opinion, how harmful is … (moist snuff, snus, e-cigarettes) to general health?’ and ‘In your opinion, to what extent does … cause cancer?’
31	Schneider et al., 2012	NA	NA	NA	18.59 (6.31)	Motivation to quit was assessed with four items: What extent the warnings induced them to: “consider ceasing their cigarette consumption”, “consider reducing their cigarette consumption”, **“think about the health risks associated with smoking”** and “refrain from smoking a cigarette at the moment”
32	Swayampakala et al., 2014	Australia	62.83	65.5	NA	“To the best of your knowledge, indicate which illness (emphysema, heart attacks, bladder cancer, blindness, impotence in male smokers, gangrene and hepatitis (non-smoking related disease), if any, are caused by smoking cigarettes?” **Note: percentages of the six risks were averaged for pre and post result**
		Canada	56.5	61	NA	
		Mexico	55.5	55.3	NA	
33	White et al., 2008	Experimental smoker	69.6	79	NA	Agreed or disagreed that smoking caused a number of different illnesses or harms (disease in toes and fingers, mouth cancer, clogs arteries, emphysema, leading cause of death). **Note: percentages of the five risks were averaged for pre and post result**
		Established smoker	67.4	78.4	NA	
34	Yong et al., 2013	NA	30.9 (2.14)	48.3 (2.16)	NA	“To what extent, if at all, do the health warnings make you think about the health risks (health danger) of smoking?”
35	Yong et al., 2016	NA	1.82	1.95	NA	Cognitive reactions combined 3 questions: “To what extent, if at all, do the warning labels make you think about the health risks of smoking?”; “To what extent, if at all, do the warning labels on cigarette packs make you more likely to quit smoking?”; “In the past 6 months, have warning labels on cigarette packages led you to think about quitting?”.

^*^Results generated online from web plot digitize

Twenty-four studies showed an increase in perceived harm over time after the implementation of GHWs [3, 5, 34, 50, 59]. Gravely, Fong [46] reported an increase of perceived risk between pre-policy wave 2 (31.5%) and post-policy wave 3 (43.3%). Similarly, Elton-Marshall, Xu [5] and Fathelrahman, Li [42] showed an increase in perceived risk from pre-policy (6.90%) and (M = 3.6, SD = 1.9) and post-policy (11.80%) and (M = 3.8, SD = 2.0) respectively, in Malaysia. However, Li, Fathelrahman [43] showed that whilst there was an increase in perceived harm in Malaysia from pre to post-policy (M = 0.07, SD = 0.06); M = 1.01, SD = 0.06), there was a continued decrease in perceived harm over the years after implementation of GHW (M = 0.47, SD = 0.06). It is noteworthy that all studies that increased perceived risks have GHWs that are 50% and greater, and most of them have credible sources listed next to the warning messages.

Furthermore, the remaining 10 studies found a decrease in perceived harm over the period of the study. As briefly introduced above, a further six studies showed a decrease over a number of years after policy implementation of graphic health or improved graphic [1, 32, 35, 58, 61, 62]. As found in Borland, Wilson [35], Canada in wave 1 had implemented its GHWs and over the four survey waves, the measure of perceived harm decreased from 1.93 (response strength) to 1.84. Hitchman, Driezen [58] confirmed that there was a decrease (OR: − 0.320) in the effectiveness of Canada’s GHWs over the period 2003–2011. Likewise, similar results were found in Australia with Kennedy, Spafford [62], investigating the impact of blindness tobacco warning labels, finding that 69.5% of Australians were aware of the risk that smoking causes blindness, however 3 y later that decreased to 57.5% of Australians. The wear-out effects seem to be evident regardless of GHW formats.

Three studies found that GHWs had a negative impact on perceived risk [36, 37, 52]. All of these studies had short exposure periods with Brewer, Parada [52] and Parada, Hall [36] only testing pre and post exposure to health warnings. Brewer, Parada [52] decreased perceived risk from 0.04 to 0.03 over the period of the study and similarly Parada, Hall [36] showed the same trend of perceived risk decreasing from 3.2 to 3.1 over the course of the four weeks. No clear conclusions can be drawn about GHW formats in relation to perceived risks.

### Quit intention outcomes

Twenty-one of the 35 studies measured the impact of GHW on people’s intention to quit smoking. In four of the studies, quit intentions and perceived risk measures were combined into a cognitive response measure [35, 38, 43, 61] therefore it is not possible to separate the effect of warning format variables on quit intentions from the effect on perceived harm variables for the combined measures. Measures used for intention to quit focused on the “extent to which health warning labels make you more likely to quit smoking” in some studies [5, 41, 46, 59]. In other studies, measures of quit intention focused around planning or intending to quit smoking in the next week, month or year [4, 45, 48, 54].

Eleven studies showed an increase in quit intentions over the course of the study [4, 5, 36, 38, 41, 45, 46, 50, 51, 53, 59]. Gravely, Fong [46] found an increase in thinking about quitting from 21.6% of respondents at pre-policy to 31.3% post-policy in India. Likewise, the implementation of policy in Mauritius [59] resulted in quit intentions increase from 13.5% pre-policy to 26.6% post-policy which was 10–12 months later. In comparison to others, Hall, Sheeran [53] was a short study comprising of a four-week trial, though it had similar findings of an increase in quit intentions over this period. Baseline measures of quit intention among participants in the pictorial warning trial were M = 2.3, SD = 0.9 and at week four, after exposure to pictorial warnings on their cigarette package, quit intentions significantly increased to M = 2.57, SD = 1.07. However, Brewer, Parada [52] followed a comparable study design over a four-week period and found a decrease in quit intentions from baseline (Cohen’s D = 0.26) to week four follow-up (Cohen’s D = 0.16).

Six of the studies identified a decrease in quit intentions after completion of the study [35, 43, 48, 52, 58, 61]. Like perceived risk, a few studies highlighted the immediate impact that new GHW have in increasing quit intentions but an overall decline in the impact months or years later [35, 43, 48, 58]. Hitchman, Driezen [58] and Borland, Wilson [35] both confirmed that for Canada there was a decrease in the impact of its GHW from when they were introduced in 2002 to 2011. Borland, Wilson [35] found that the strength of response for quit intentions (Range from 1 to 3.67) decreased from 1.93 in Wave 1 (2002) to 1.84 in Wave 4 (2006). Similarly, Hitchman, Driezen [58] reported that quit intentions decreased by − 0.504 (OR) over an eight-year period. Durkin, Brennan [48] found a related pattern in Australia. Prior to the introduction of plain packaging 36.10% of smokers intended to quit. During the last stage of the study, quit intentions increased to 42%. However, one-year post implementation of the plain packaging quit intentions decreased back to 35.60%. Taken together, studies identify a positive impact of GHW on intentions to quit that can decline over time. Table 8 presents detailed findings from each relevant study.

**Table 8:** Quit intention outcomes

No.	Author(s), publication year	Group	Quit Intentions		OR or Beta	Measures
			Pre	Post		
2	Borland et al., 2009*	Australia	1.68	2.04	NA	Cognitive responses combined two questions: “Extent to which the warnings both made the respondent think about the health risks of smoking” and “made them more likely to quit smoking”
		UK	1.95	1.81	NA	
		Canada	1.93	1.84	NA	
3	Brewer et al., 2016	NA	2.3 (0.9)	2.7 (1.0)	NA	Quit intentions combined three questions: “How likely are you to quit smoking in the next month?”, “How much do you plan to quit smoking in the next month?” and “How interested are you in quitting smoking in the next month?”
4	Brewer et al., 2019	NA	0.26 (0.18, 0.35)	0.16 (0.07, 0.24	NA	Quit intentions combines 3 questions: “How interested are you in quitting smoking in the next month?”, “How likely are you to quit smoking in the next month?” and “Are you planning to quit smoking”
6	Durkin et al., 2015	Pre PP	NA	NA	36.10% (OR:1.00)	“Do you intend to quit in the next month?”
		Late transition	NA	NA	42% (OR: 1.42)	
		Post 1 year PP	NA	NA	35.60% (OR: 0.98)	
7	Elton-Marshall et al., 2015	NA	9.0%	18.0%	NA	“To what extent, if at all, do the health warnings on cigarette packs make you more likely to quit smoking?”
8	Fathelrahman et al., 2010	NA	30 (42.9%)	28 (40.0%)	NA	“Planning to quit smoking in the future (within the next month, within the next 6 months, sometime in the future beyond six months or not planning to quit)”
9	Fathelrahman et al., 2013	NA	418 (41.1%)	239 (29.8%)	NA	“any interest in quitting”
11	Gravely et al., 2016a	NA	20.6	31.3	OR: 1.76	“To what extent do the health warnings on cigarette packs make you think about quitting smoking?”
12	Gravely et al., 2016b	NA	19.8% (95% CI 14.6; 26.4)	20.5% (95% CI 15.2; 27.0)	NA	“Are you planning to quit using smokeless tobacco”: Within the next month; Within the next 6 months; Sometime in the future beyond 6 months; Not planning to quit
13	Green et al., 2014	NA	13.50%	26.60%	OR: 2.69 (95% CI = 1.75–4.15)	“To what extent, if at all, do the warning labels on cigarette packs make you more likely to quit smoking”
15	Hall et al., 2018	NA	2.3 (0.9)	2.57 (1.07)	NA	Quit intentions combined 3 questions: “How much do you plan to quit smoking in the next month?”, “How interested are you in quitting smoking in the next month?” and “How likely are you to quit smoking in the next month?”
16	Hitchman et al., 2014	NA	NA	NA	OR: −0.504 (×2 = 6.48)	“To what extent, if at all, do the warning labels on cigarette packs make you more likely to quit smoking”
19	Li et al., 2015	NA	2.1	1.9	NA	Cognitive response combined 3 questions: “made them think about the health risks of smoking”, “made them more likely to quit smoking” and “if ‘warning labels on cigarette packages’ motivated them to think about quitting in the past 6 months”
20	Lin et al., 2016	Thailand	0.49 (0.06)	0.61 (0.06)	NA	Cognitive response combined 2 questions: “made them think about the health risks of smoking” and “made them more likely to quit smoking”
		Malaysia (wave 2–4)	0.07 (0.06)	1.01 (0.06)	NA	
		Malaysia (wave 4–6)	1.01 (0.06)	0.47 (0.06)	NA	
23	McQueen et al., 2015	NA	130 (71%)	133 (73%)	NA	“made them think about quitting”
24	Nagelhout et al., 2016	UK	NA	NA	OR: 1.34	Cognitive responses combined 3 questions: “To what extent, if at all, do the warning labels make you think about the health risks of smoking?”, “To what extent, if at all, do the warning labels on cigarette packs make you more likely to quit smoking?” and “In the past 6 months, have warning labels on cigarette packages led you to think about quitting?”
		France	NA	NA	OR: 0.7	
26	Nicholson et al., 2017	NA	50	54	NA	“Perceive warning labels effective to quit or stay quit”
27	Osman et al., 2016	NA	NA	NA	OR: 1.21	“Their intention to quit smoking”
28	Parada et al., 2017*	NA	2.4 (mean)	2.6	NA	Three questions: “How interested are you in quitting smoking in the next month,” “How much do you plan to quit smoking in the next month?” and “How likely are you to quit smoking in the next month?”
31	Schneider et al., 2012	NA	NA	NA	18.59 (6.31)	Motivation to quit was assessed with four items: What extent the warnings induced them to: **“consider ceasing their cigarette consumption”**, “consider reducing their cigarette consumption”, “think about the health risks associated with smoking” and “refrain from smoking a cigarette at the moment”
34	Yong et al., 2013	NA	27.9 (2.37)	42.0 (2.19)	NA	“To what extent, if at all, do the health warnings on cigarette packs make you more likely to quit smoking?”

^*^Results generated online from web plot digitize

## Discussion

GHWs are an important component of the suite of tobacco control policies [63–65]. Literature has identified that a significant wear out becomes present when GHW are left in market over sustained periods [36, 66, 67]. To contribute to the evidence base, this review aimed to systematically identify and synthesize evidence from longitudinal studies which tested different GHW formats and message frames to understand the effects on perceived risk of smoking and benefits of quitting and intentions to quit smoking. Taken together, a total of 35 studies demonstrated that GHWs increase awareness of health risks over time and they have desired outcomes including changing beliefs about smoking, increasing intention to quit and many more. The current review delved deeper into the mechanics of GHWs relationship to perceived health risks and intentions to quit smoking. The contributions of this study are fourfold. Each contribution is detailed in turn.

Firstly, the review identified that pictorial GHWs deliver a superior performance when compared to text only messages both in terms of magnitude and the number of positive outcomes achieved. Pictorial GHWs that are prominent (bigger than 50%) increase perceived health risks and intentions to quit smoking. This review found that GHWs exceeding 50% or more of pack size were most common, and most GHWs were printed on both the front and the back of packs. This review also identified that all studies that increased perceived health risks featured GHWs that exceeded 50% or more of cigarette pack size. The implications are clear that any country that has not mandated that GHWs be 50% of the pack or more should move to do so, given that health risks and intentions to quit smoking can result. Studies have called for more distribution channels of GHWs beyond tobacco packaging [68, 69]. Four studies [3, 33, 47, 57] in this review used package inserts as a complimentary approach to traditional GHWs. Other studies also call for GHWs to be displayed at the point-of-sale [70], via national TV ads [71] and digital channels [68]. Schmidt, Ranney [69] identified that GHWs in public service announcements can improve GHW persuasion.

Secondly, this study illuminates an ongoing reliance on negatively framed messages. Support for the use of positive message framing when targeting illness prevention behaviors including smoking cessation is available [13]. Evidence demonstrates that smokers react to negative messages (fear appeals) harming the quit intention relationship [12]. This review identifies a landscape dominated by negatively framed messages and whilst this approach is effective in increasing understanding of the risks smokers face, other approaches may be needed to induce smokers who are fully aware of health risks to quit smoking. Examination of the wider message framing evidence base indicates that positively framed messages can invoke behavioral change [72, 73]. Accordingly, research examining positive framing is called for to identify messages that can be utilized to increase quit intentions and quitting behavior. This systematic literature review has identified there is no work utilizing a longitudinal research design that has examined the positive benefits of quitting indicating there is a need to extend understanding of the role communication of the benefits of quitting (saving money, having more energy) has on quitting intentions. Further, most of the GHWs are fear-based images with strong visual and direct stimuli and only a few studies have symbolic images. The usage of symbolic images or more subtle cues needs to be studied further to draw conclusive evidence.

Thirdly, this review unveiled that source attribution is not common, although research has indicated credibility strongly contributes to believability [69, 74–76]. Only half of the studies specified credible sources for their warning messages (e.g., Health Canada or US HHS). Perceived risks are a cognitive measure that is heavily influenced by trustworthiness of expertise [77] and perceived authoritativeness [78]. Schmidt, Ranney [69] argued that source credibility needs to be further investigated, either topic-specific, or organization-specific, in order to improve long-term behavioral change in response to tobacco control communications.

Lastly, this review also identified that perceived harms and quit intentions were generally increased by GHWs, and confirmed prior literature [32, 43], noting that wear-out was observed across a range of GHW formats. While understanding the role of GHW formats and message framing must remain a priority, research focused on identifying GHW wear out points is needed to ensure GHW efficacy is optimized. Practically, changing sets of GHWs at the beginning of wear-out is the key to maximize the effectiveness.

### Limitations and Future Research

This review has three major limitations that also warrant future research to further extend our understanding of the relationship between GHW formatting and the effectiveness of GHWs. Firstly, outcome measures are inconsistent, which needs to be considered when interpreting the findings in this paper. Future research should focus on using widely accepted and validated scales (such as scales from the Measurement Instrument Database for the Social Science, https://www.midss.org/) in order to produce results that can be directly compared across studies. Secondly, most evaluation work uses non-randomized study designs. Due to the nature of GHW policy implementation, confounding issues become inevitable. More experimental studies conducted in highly controlled environments should be conducted to deliver optimal levels of scientific rigor. RCT studies were generally assessed with a strong quality rating; However, RCTs tend to have smaller sample sizes and shorter study timeframes, which should be taken into consideration when interpreting review findings. Future research should consider utilizing RCT’s as the default study design and increase sample sizes if resources permit, in order to achieve the most reliable results. Lastly, this review did not include any grey literature such as government reports, policy statements, and industry data reports. Although grey literature was generally deemed as low-quality, in this review a thorough check was conducted in the attempt to identify any relevant grey literature in recent years, and none was found and included. Future research should consider widening the types and years of grey literature in order to capture any insights to further extend our understanding of the relationship between GHW formatting and the effectiveness of GHWs.

## Conclusions

This review focuses on the strongest evidence available in the literature. Longitudinal research designs permit causal conclusions on the format of GHWs and message framing on quit intentions and smoking risks to be drawn. The findings of this review contribute to the literature extending understanding of GHW formats identifying that negatively framed pictorial GHW warnings displayed on 50% or more of packs increase perceived health risks and intentions to quit smoking. An ongoing reliance on negatively framed messages was evident, which is concerning given these can create reactance for some smokers and testing of alternate approaches (e.g. benefits of quitting smoking) is recommended given the capacity for positively framed messaging to achieve behavior changes. This review recommends further testing of alternative imagery warnings that can better communicate benefits of quitting and more use of objective measures to extend examinations beyond self-reporting.

## Supplementary Information

**Additional file 1:** PRISMA 2009 Checklist.

## Data Availability

All data generated or analysed during this study are included in this published article [and its supplementary information files].

## Abbreviations

FDA: Food and Drug Administration (US)
DoH: Department of Health
GHW: Graphic Health Warning
NRSI: Non-randomized studies of interventions
PP: Plain Packaging
RCTs: Randomized controlled trials
RoB2: Risk of Bias tools 2 [31]
ROBINS-I: Risk Of Bias In Non-Randomized Studies - of Interventions [30]
RYO: Roll-Your-Own cigarettes
